# Mathematical modeling of human memory

**DOI:** 10.3389/fpsyg.2023.1298235

**Published:** 2023-12-22

**Authors:** Paolo Finotelli, Francis Eustache

**Affiliations:** Normandie Univ, UNICAEN, PSL Université Paris, EPHE, INSERM, U1077, CHU de Caen, Centre Cyceron, Neuropsychologie et Imagerie de la Mémoire Humaine, Caen, France

**Keywords:** memory, mathematics, amnesia, models, neuropsychology, demantia

## Abstract

The mathematical study of human memory is still an open challenge. Cognitive psychology and neuroscience have given a big contribution to understand how the human memory is structured and works. Cognitive psychologists developed experimental paradigms, conceived quantitative measures of performance in memory tasks for both healthy people and patients with memory disorders, but in terms of mathematical modeling human memory there is still a lot to do. There are many ways to mathematically model human memory, for example, by using mathematical analysis, linear algebra, statistics, and artificial neural networks. The aim of this study is to provide the reader with a description of some prominent models, involving mathematical analysis and linear algebra, designed to describe how memory works by predicting the results of psychological experiments. We have ordered the models from a chronological point of view and, for each model, we have emphasized what are, in our opinion, the strong and weak points. We are aware that this study covers just a part of human memory modeling as well as that we have made a personal selection, which is arguable. Nevertheless, our hope is to help scientists to modeling human memory and its diseases.

## 1 Introduction

In neuropsychology, memory is conceived as a complex function made up of several interacting systems. Five major systems are most often differentiated: working memory (or short-term memory), episodic memory, semantic memory, perceptual memory, and procedural memory. These different systems, which make up individual memory, interact with collective memory. Memory makes it possible to record, store, and restore information, but this definition is incomplete in view of its complexity since it forges our identity, constitutes the source of our thoughts, operates back and forth with representations of our personal and collective past, projects them toward an imagined future, builds our life trajectory, and participates in the regulation of our social relations and our decision-making. Amnesic syndromes as well as dementia syndromes has been the main sources of inference to differentiate several forms of memory, by highlighting dissociations between disturbed and preserved memory capacities in these pathologies. Regarding the interactive construction of memory systems and processes, we refer to the Memory NEo-Structural Inter-Systemic model (MNESIS), which is a macromodel based on neuropsychological data. The reader can find all the details in Eustache et al. (2016).

### 1.1 Working memory

Working memory is the memory system responsible for temporarily maintaining and manipulating information needed to perform activities as diverse as understanding, learning, and reasoning. It consists of two satellite storage systems (the phonological loop and the visuo-spatial notebook), supervised by an attentional component, the central administrator.

The phonological loop is responsible for storing verbal information, manipulating it, and refreshing it. The visuospatial notebook is involved in the storage of spatial and visual information as well as in the formation and manipulation of mental images. The central administrator manages the transfer of information to long-term memory. It relies on an episodic buffer, responsible for the temporary storage of integrated information from different sources, which plays a role in encoding and retrieval in episodic memory. It is thus at the interface between several systems and uses a multidimensional code common to these different systems.

### 1.2 Long-term memory

Within long-term memory, episodic memory is the memory of personally experienced events, located in their temporal-spatial context of acquisition. Its fundamental characteristic is to allow the conscious memory of a previous experience: The event itself (what), but also the place (where) and the moment (when) it occurred. The retrieval of a memory in episodic memory gives the impression of reliving the event due to a “mental journey in time” through one's own past, associated with “autonoetic awareness” (or self-awareness).

Semantic memory is the memory of concepts, knowledge about the world, regardless of their context of acquisition. It is associated with “noetic consciousness” or awareness of the existence of objects and various regularities. Semantic memory allows introspective behavior about the world but also includes general knowledge about oneself: personal semantics.

Representations can thus be based on general (semantic type) or specific (episodic type) knowledge. On the contrary, procedural memory makes it possible to acquire skills, with training (over many trials), and to restore them without referring to previous experiences. It is expressed in action and its contents are difficult to verbalize. Procedural memory allows us to perform activities without explicitly remembering the procedures and without awareness of when we learned them.

Another distinction opposes explicit memory and implicit memory. Explicit memory refers to situations in which a subject voluntarily recalls information. On the contrary, implicit memory is brought into play without the subject's knowledge, when a previous experience modifies his performance in a task that does not require his conscious recall. Thus, the fact of seeing an image for the first time facilitates its subsequent identification, including if it is presented in a degraded form. Implicit memory depends on the system of perceptual representations, which corresponds to a perceptual memory and makes it possible to maintain information in memory, even if it is meaningless, and can manifest itself without the knowledge of the subject.

**Figure 1:**
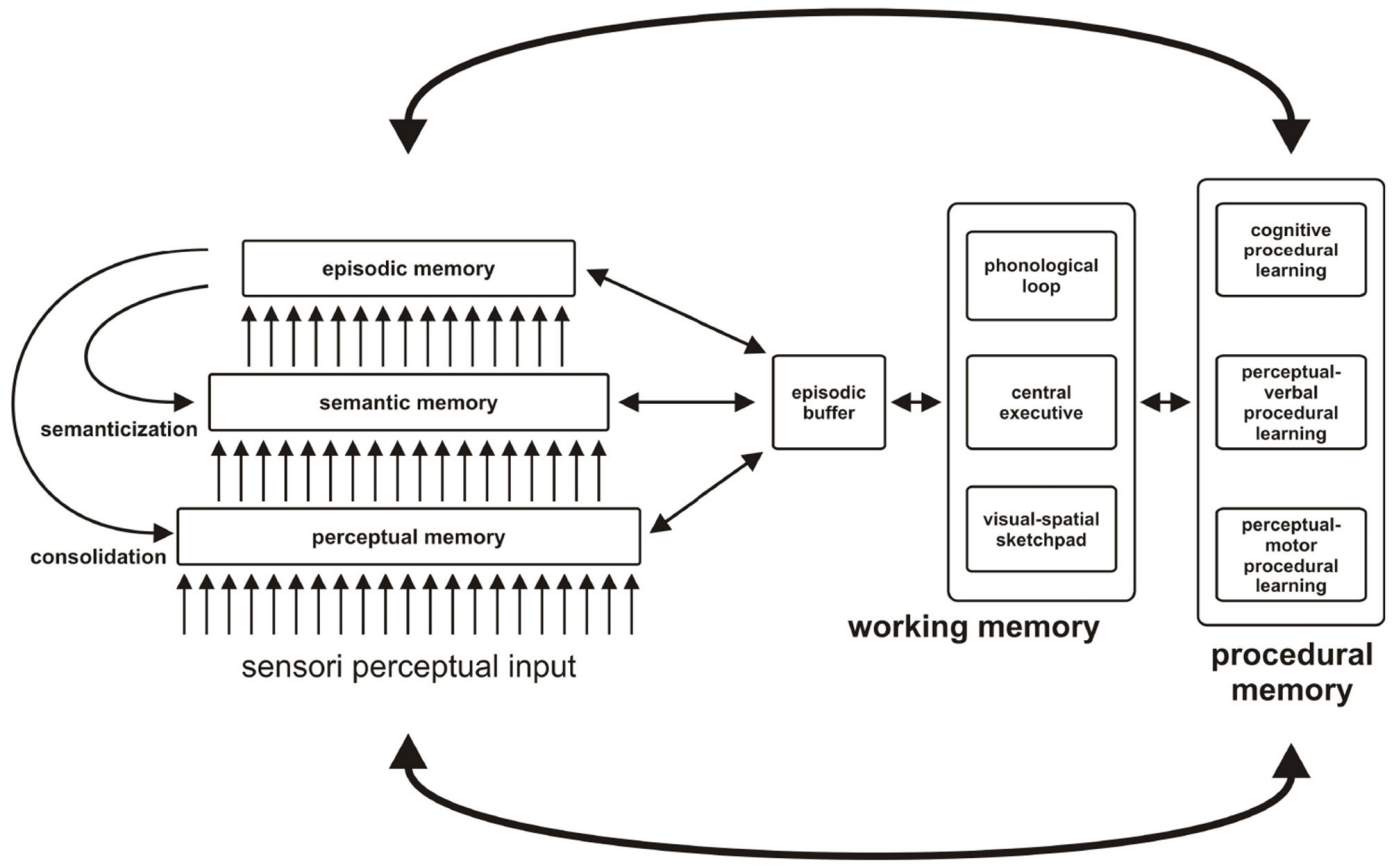
MNESIS, an overall representation of individual memory, and its interface with collective memory. MNESIS represents the five systems of individual memory. The three long-term representation systems (perceptual memory, semantic memory, and episodic memory) are organized hierarchically. Many episodic memories undergo a process of semantization over time. In addition, the phenomena of reviviscence, both conscious and unconscious, are essential for mnesic consolidation, thus underlining the importance of the dynamic and reconstructive nature of memory. This characteristic of memory has as its corollary the modification of the memory trace and the possible formation of false memories. At the center of the MNESIS model, there is the working memory, with the classic components (the central administrator, the phonological loop, and the visuo-spatial notebook) and the episodic buffer, a temporary interface structure that solicits different neurocognitive systems. Depending on the activity in progress, it can regulate the expression of self-awareness in the present or participate in the establishment of a new skill. Procedural memory is presented, with a hierarchy ranging from the support of motor and perceptual-motor skills to that of cognitive skills. The links with perceptual memory are favored for perceptual-motor procedural memory and with declarative systems for cognitive procedural memory. In any case, interactions with representation systems (including working memory) are particularly important during the procedural learning phase. The bonds loosen during the progressive automation of learning (adapted from Eustache et al., 2016).

The MNESIS model (Eustache et al., 2016) specifies the interactive functioning of memory systems, which take their place within collective memory, see Figure 1.

## 2 Mathematical models of human memory

This section is dedicated to illustrating the most theoretical important mathematical models of human memory present in the literature, which are based on concepts proper to mathematical analysis and linear algebra, such as mathematical analysis, differential equations, vector, and matrix algebra. The literature on mathematical and computational models of memory is vast (see for example, Sun, 2008). Hence, we focus our review just on models whose rationale is underpinned by mathematical analysis as well as linear algebra. With “analysis” we mean the branch of mathematics dealing with continuous functions, limits, and related theories, such as differentiation, integration, measure, infinite sequences, series, and analytic functions. Differential equations are an important (sub)area of mathematical analysis with many applications in the study of memory, and more broadly of the brain. Differently, linear algebra deals with vectors and matrices and, more generally, with vector spaces and linear transformations. From this perspective, the history of attempts to model memory dates back to the late 1800s and continues to our days. Interestingly, after the approaches of pioneers in the study of memory such as Ribot (1906) and Ebbinghaus (1913), there was a period of stalemate, a sort of “memory modeling winter” which gained momentum starting from the 60 of the last century, due to an increasing interest and to new computational tools, it is becoming more and more popular.

### 2.1 Ebbinghaus forgetting curve

The study of higher mental processes by using experimentation started in the second part of the 19^*th*^ century due to Ebbinghaus, such an approach was in opposition to the popularly held thought of the time. In 1885, Ebbinghaus, in his groundbreaking *Memory. A Contribution to Experimental Psychology* (original title: Über das Gedächtnis) described the experiments he conducted to describe the processes of forgetting (and learning). His experiments represent one of the first attempts to study the mechanisms of forgetting even if he used himself as the sole subject. Indeed, in his experiment, he memorized lists of three letter nonsense syllable words–two consonants and one vowel in the middle. Then, he measured his own capacity to relearn a given list of words after a variety of given time period. He found that forgetting occurs in a systematic manner, beginning rapidly and then leveling off. He plotted out his results diving rise to the famous *Ebbinghaus forgetting curve*. Ebbinghaus remarked that first, much of what it is forgotten is lost soon after it is originally learned. Second, the amount of forgetting eventually levels off.

Many equations have since been proposed to approximate forgetting. For example, in 1985, Loftus (1985) described a new method for determining the effect of original learning (or any other variable) on forgetting. Loftus tried to answer a major question, i.e., how much forgetting time is required for memory performance to fall from any given level to some lower level? If this time is the same for different degrees of original learning, then forgetting would not be affected by degree of original learning. In terms of evaluation, if this time is greater for higher degrees of original learning, then forgetting is slower with higher original learning. Loftus applied his method to a variety of forgetting data, the outcomes indicated that forgetting is slower for higher degrees of original learning. Loftus supposed that forgetting is characterized by the following assumptions: First, original learning produces some amount of information in memory. The higher the original learning, the greater the amount of information. Second, following learning, the amount of retrievable information decays exponentially over time. Third, performance, i.e. number of items recalled or recognized, is a linear function of information. If *P* is the performance (e.g., number of items recalled), which Loftus assumed to be equal to the amount of information at time *t* following learning, then it is possible summarize the model by means of the following equation:


(1) $$\begin{array}{l} P\left(t\right)=\varrho e^{\varsigma t} \end{array}$$

where ϱ represents the units of information are originally stored in memory, while ς the rate of decay. In conclusion, Loftus remarked that the application of the proposed method to a variety of forgetting data indicated that forgetting is slower for higher degrees of original learning.

In a similar way, 10 years later, in 1995, Wozniak et al. (1995), proposed perhaps the simplest forgetting curve, being an exponential curve described in by the Equation (2). The main characteristic of such a proposal is the existence of two components of long-term memory.


(2) $$\begin{array}{l} R=e^{-\frac{t}{S}} \end{array}$$

where *R* is retrievability (a measure of how easy it is to retrieve a piece of information from memory) and *S* is stability of memory (determines how fast *R* falls over time in the absence of training, testing, or other recall), and *t* is time.

As a final observation, around the same time, Ebbinghaus developed the forgetting curve, psychologist Sigmund Freud theorized that people intentionally forgot things in order to push bad thoughts and feelings deep into their unconscious, a process he called “repression.” There is debate as to whether (or how often) memory repression really occurs (McNally, 2004).

#### 2.1.1 Strong and weak points of Ebbinghaus' work on memory

##### 2.1.1.1 Strong points

- It was a pioneering study.
- The model served as a model for further studies on cognitive abilities and psychological evaluations.

##### 2.1.1.2 Weak points

- Ebbinghaus was the only subject in the study, and therefore, it was not generalizable to the population. In addition, a large bias is to be expected when a subject is a participant in the experiment as well as the researcher.
- There are other analytical forms of the forgetting curve that could fit the obtained result, for example, the power law (see Wixted and Ebbesen, 1991). Nevertheless, the exponential form has several applications and in other brain-related fields such as complex brain network analysis, where the probability of formation of links follows such an analytical form.

#### 2.1.2 Mathematical developments

A remarkable development (and implementation, too) of Ebbinghaus' theory is the study by Georgiou et al. (2021). Basically, Georgiou, Katkov, and Tsodyks proposed a model which is strength-dependent retroactive interference between the memories. Hence, only if a stronger memory is acquired after the weaker one, then the weaker one is erased. The model results in powerlaw retention curves with exponents that very slowly decline toward -1. The asymptotic value for all realistic time lags that can be measured experimentally.

### 2.2 Ribot's law

In 1906, Ribot in his book *Les maladies de la mémoire* described the so called Ribot's law of retrograde amnesia (actually it was hypothesized in 1881 by Théodule Ribot itself). Such a law states that there is a time gradient in retrograde amnesia, so recent memories are more likely to be lost than the more remote memories. We remark that not all patients with retrograde amnesia report the symptoms of Ribot's law.

In other words, the Ribot gradient is a pattern where memory loss in retrograde amnesia is larger for recent periods rather than for remote periods. A possible explanation for this gradient lies in the consolidation of memories, which is more prominent in long-term memories. Consolidation is a key concept to explain the gradient in retrograde amnesia. For example, if the hippocampal memory system is damaged in a subject, she/he will tend to lose more of their recent than of their remote memories (Kopelman, 1989; Squire, 1992). That is exactly the Ribot gradient! Ribot, basically, suggested that recent memories might be more vulnerable to brain damage than remote memories.

If we assume that the retrieval of memories depends on the hippocampal memory system then the Ribot gradient can be intuitively interpreted. In this sense, consolidation is a fundamental process. Indeed, through consolidation, memories gradually become stored in the neocortex, giving rise to the corticohippocampal system, making them independent of the hippocampal system (Squire et al., 1984; Squire and Alvarez, 1995). If the hippocampal system is damaged, recent memories are lost because they still depend on such a system. Differently, since old memories have already been stored in the neocortex through consolidation, they are thus spared. It is possible to provide the analytical form of the Ribot gradient, as shown in Murre et al. (2013). If we refer to *r*_1_(*t*) as to the intensity of the hippocampal process (as a function of time) and to *r*_2_(*t*) as to that of the neocortical process, then the sum of the intensities of the individual processes *r*(*t*) = *r*_1_(*t*)+*r*_2_(*t*) represents the total memory intensity (see for example, Memory Chain Model Murre and Chessa, 2011). This superimposition of intensities allows to treat specific pathological cases. For example, a full lesion at time *t*_*l*_ of the hippocampus cause the removal of the term *r*_1_(*t*_*l*_) from the total intensity *r*(*t*_*l*_). As a consequence, the only remaining term is *r*_2_(*t*_*l*_), the neocortical intensity at the time of the lesion, *t*_*l*_, which reflects the result of the consolidation process until the lesioning time *t*_*l*_. Hence, it follows that the shape of the Ribot gradient with a full hippocampal lesion at time *t*_*l*_ is identical to the expression for *r*_2_(*t*_*l*_). The predicted shape of these test gradients is, therefore, given by


(3) $$\begin{array}{l} p_{Ribot}\left(t\right)=1-e^{-r_{2}\left(t_{l}\right)} \end{array}$$

We remark that tests of retrograde amnesia do not measure intensity directly, but they rather measure recall probability, that is, the reason for the symbol *p*_*Ribot*_(*t*), *p* stands for “probability”.

#### 2.2.1 Strong and weak of Ribot's law

##### 2.2.1.1 Strong points

- Similarly to what written for the Ebbinghaus' works, the Ribot's law was a pioneering and leading study.
- The model served as a model for further studies on cognitive abilities, psychological evaluations as well as to investigate memory diseases.
- Many neurodegenerative diseases, including Alzheimer's disease, are also linked to retrograde amnesia and consequently can be explained, at least as a first approximation, by the Ribot's law.

##### 2.2.1.2 Weak points

- Currently, Ribot's law is not universally accepted as a supporting example for memory consolidation and storage. As a component of the standard model memory of systems consolidation, it is challenged by the multiple trace theory which states that the hippocampus is always activated in the storage and retrieval of episodic memory regardless of memory age.
- Similarly to what is observed for the Ebbinghaus' curve, there are other analytical forms that could well explain Ribot's law (see Wixted and Ebbesen, 1991) even though the exponential form has some properties very useful to take advantage of for modeling purposes.

#### 2.2.2 Mathematical developments

In our opinion, Murre et al. (2013) showed a stunning example of an application of Ribot's law to modeling amnesias. Their model assumes that memory processes can be decomposed into a number of processes that contain memory representations. Memory processes has a wide range of variability, from milliseconds (extremely short-term processes) to decades (very long-term processes). A memory representation could be thought of as consisting of one or more traces, such a representation can be viewed as neural pathways, any of which suffices to retrieve the memory. This trace generation is governed in a random way. Each trace in a process generates traces of its representation in the next higher process, for example, through long-term potentiation (LTP) in the hippocampus (Abraham, 2003) or neocortex (Racine et al., 1995). LTP is a stable facilitation of synaptic potentials after high-frequency synaptic activity, is very prominent in the hippocampus and is a leading candidate memory storage mechanism. We remark that a trace can be overwritten by different traces or by neural noise; in these cases, the trace is lost. As a consequence, it can no longer generate new traces in higher processes. The authors hypothesize that first, all traces in a process share the same loss probability; second, higher processes in the chain have lower decline rates. If the hippocampus undergoes a lesion at time *t*_*l*_, then no more memories will be formed after that. In addition, no more consolidation from hippocampus-to-cortex happens.

If *r*(*t*_*l*_) denotes the intensity of a particular memory at the time of the lesion, after *t*_*l*_, a decline of the memory intensity, with neocortical decline rate *a*_2_, will be observed, the equation representing this case is given by


(4) $$\begin{array}{l} r\left(t_{l}\right)e^{-a_{2}\left(\tau \right)} \end{array}$$

where τ is the time elapsed since the lesion. Interestingly, the authors introduce the case partial lesion of the hippocampus, this means that they leave the size of the lesion as a free parameter. The lesion parameter is denoted as λ, λ ranges from 0 to 1, extremes included. If the lesion parameter is 0, no lesion is present; on the opposite, if λ = 1, there is a complete lesion. In case of a partial lesion, the Ribot gradient is equal to


(5) $$\begin{array}{l} p_{Ribot}\left(t\right)=1-e^{-\left[\left(1-\lambda \right)r_{1}\left(t\right)+r_{2}\left(t\right)\right]} \end{array}$$

This is the most general form of the model, based on the Ribot gradient, proposed by the authors. Generally, the tests of retrograde amnesia provide recall probabilities as a function of time elapsed, such a probability is denoted as *p*(*t*). Mathematically speaking, an observed recall probability *p*(*t*) can be transformed into an intensity *r*(*t*) by taking − ln(1 − *p*(*t*)), where ln is the natural-based logarithm.

### 2.3 Atkinson-Shiffrin memory model

**Figure 2:**
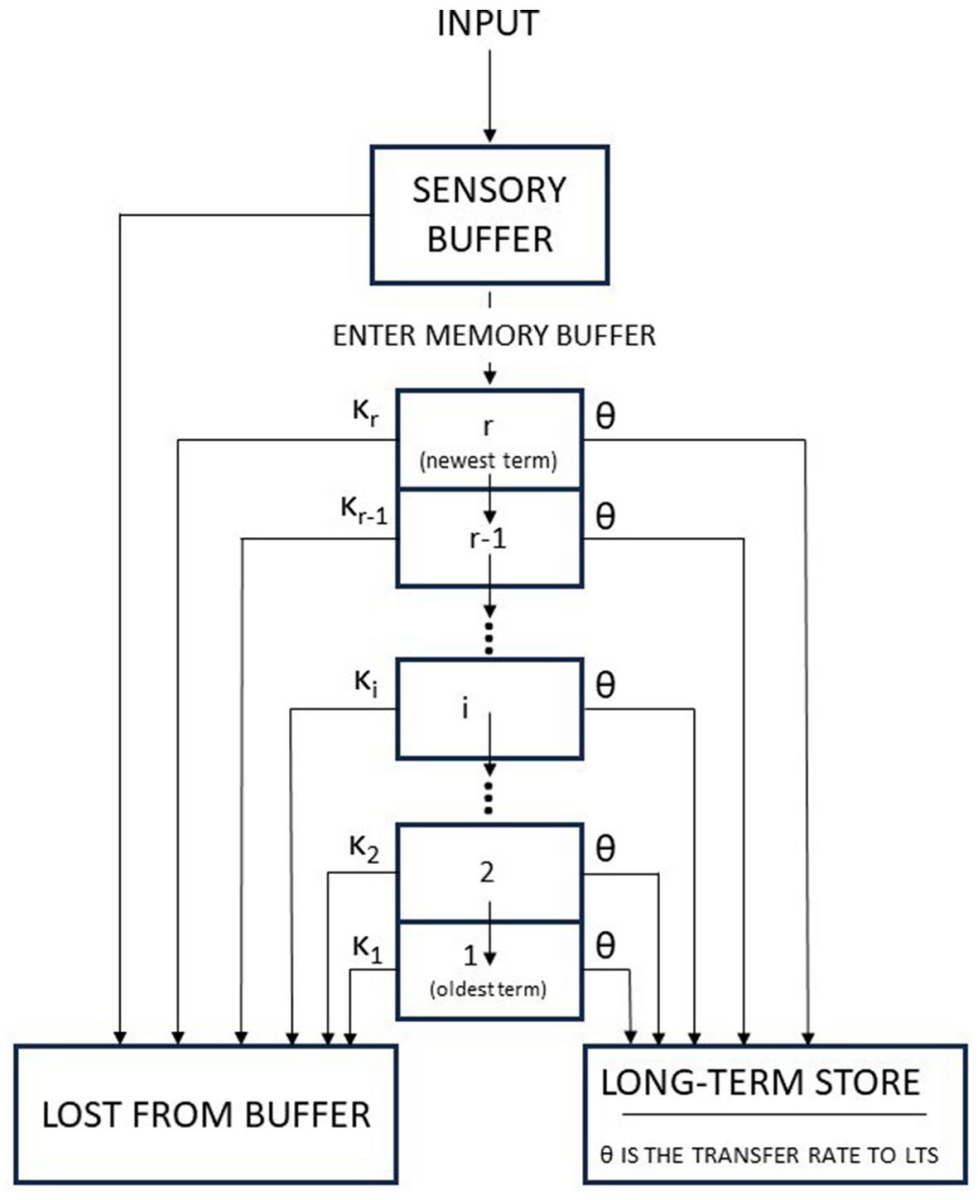
The Atkinson-Shiffrin memory model: the flow chart characterizing inputs to the memory system (adapted from Atkinson et al., 1967).

The Atkinson-Shiffrin model (also known as the multi-store model or modal model) is a model of memory proposed in 1968 by Atkinson and Shiffrin (1968). Such a model is very influential. This model asserts that human memory has three separate components: First, a sensory register, where sensory information enters memory. Second, a short-term store, also called short-term memory (STM), which receives and holds input from both the sensory register and the long-term store. Third, a long-term store, where information which has been rehearsed (explained below) in the short-term store is held indefinitely (see Figure 2).

#### 2.3.1 Sensory memory

The sensory memory store has a large capacity, but for a very brief duration, it encodes information from any of the senses (principally from the visual and auditory systems in Humans), and most of the information is lost through decay. The threshold above mentioned is strictly linked to the attention. Indeed, attention is the first step in remembering something; if a person's attention is focused on one of the sensory stores, then the data are likely to be transferred to STM (for more details, see for example Goldstein, 2019).

#### 2.3.2 Short-term memory

If the information passes the selection in the first stage (sensory memory) of selection, then it is transferred to the short-term store (also short-term memory). As with sensory memory, the information enters short-term memory decays and is lost, but the information in the short-term store has a longer duration, approximately up to 30 s when the information is not being actively rehearsed (Posner, 1966). A key concept in this model is the memory rehearsal, and it is a term for the role of repetition in the retention of memories. It involves repeating information over and over in order to get the information processed and stored as a memory.

It should be noted that a (continuous) rehearsal acts as a sort of regeneration of the information in the memory trace, thus making it a stronger memory when transferred to the long-term store (see Section 2.3.3). Differently, if maintenance rehearsal (i.e., the repetition of the information) does not occur, then information is forgotten and lost from short term memory through the processes of displacement or decay. Once again, a thresholding procedure occurs.

In terms of capacity, the short-term store has a limit to the amount of information that it can held in, quantitatively from 5 to 9 chunks (7 ± 2).^1^

#### 2.3.3 Long-term memory

The long-term memory is in theory a sort of unlimited store, where the information could have a permanent duration. In the authors' model, the information that is stored can be transferred to the short-term store where it can be manipulated.

Information is postulated to enter the long-term store from the short-term store after the thresholding process. As Atkinson and Shiffrin modeled it, transfer from the short-term store to the long-term store is occurring for as long as the information is being attended to in the short-term store. The longer an item is held in short-term memory, the stronger its memory trace will be in long-term memory. Atkinson and Shiffrin based their observations on the studies by Hebb (1961) and Melton (1963), which show that repeated rote repetition enhances long-term memory. There is also a connection with the Ebbinghaus' studies on memory that shows how forgetting increases for items which are studied/repeated fewer times (Ebbinghaus, 1913).

Remarkably, simple rote rehearsal is not the stronger encoding processes; indeed, in author's opinion, the new information to information which has already made its way into the long-term store is a more efficient process.

The authors used a mathematical description of their proposal. Such a mathematical formalization is well detailed in Atkinson et al. (1967). In short, the memory buffer may be viewed as a state containing those items which have been selected from the sensory buffer for repeated rehearsal. Once the memory buffer is filled, each new item which enters causes one of the items currently in the buffer to be lost. It is assumed that the series of study items at the start of each experimental session fills the buffer and that the buffer stays filled thereafter. The size of the memory buffer is denoted by *r*, which is defined as the number of items which can be held simultaneously, depends upon the nature of the items and thus must be estimated for each experiment. It is also assumed that a correct response is given with probability one if an item is in the buffer at the time it is tested. Every item is selected by the sensory buffer (namely, it undergoes a thresholding process) to be entered into the memory buffer. The authors assume that the items are examined at the time they enter the sensory buffer. The items can be already in the buffer, i.e., their stimulus member can already be in the buffer or their stimulus member can not currently be in the buffer. The former case is denoted by the Authors as *O-item* (or “old” item), while the latter as *N-item* (“new” item). When an O-item is presented for study, it enters the memory buffer with probability one; the corresponding item, which was previously in the buffer, is discarded. When an N-item is presented for study, it enters the buffer with probability α, such a probability is function of the particular scheme that a subject is using to rehearse the items currently in the buffer. It is an N-item enter, the probability that such an event occur is α, then some item currently in the buffer is lost. Of course, the probability that an N-item fails to enter the buffer is 1 − α; in this case, the buffer does not undergo any change and the item in object decays and is permanently lost from memory.

The memory buffer is arranged as a push-down list. The newest item that enters the buffer is placed in slot *r*, and the item that has remained in the buffer the longest is in slot 1, i.e., the slot where the oldest item is. If an O-item enters slot r, the corresponding item is lost. Then, the other items move down one slot if necessary, retaining their former order. When an N-item is presented for study and enters the buffer (with probability α), it is placed in the *r*^*th*^ slot. The item currently in slot *j* has a probability κ_*j*_ to be discarded (or knocked out, the term used by the authors), the following condition must hold: κ_1_ + κ_2_ + κ_3_ + ... + κ_*j*_ + ...κ_*r*_ = 1, with *r* ≥ *j*. When the *j*^*th*^ item is discarded, each item above the *j*^*th*^ moves down one and the new item enters the *r*^*th*^ slot. The simplest form of κ_*j*_ is $\kappa_{j}=\frac{1}{r}$; in this case, the item to be knocked out is chosen independently of the buffer position.

At this point, let us focus on long-terms storage (LTS).

LTS can be viewed as a memory state where the information accumulates for each item. The authors made a few assumptions:

1. Information about an item may enter LTS only during the period that an item resides in the buffer.
2. The status of an item in the buffer is in no way affected by transfer of information to LTS.
3. Recall from the buffer is assumed to be perfect, and recall from LTS is not necessarily perfect and usually will not be.
4. The information is transferred to LTS at a constant rate θ during the entire period in which an item resides in the buffer; θ is the transfer rate per trial. Hence, if an item remains in the buffer for exactly *j* trials, then that item accumulated an amount of information equal to *jθ*.
5. Each trial following the trial on which an item is discarded by the buffer, then it causes a decrease of information stored in LTS by a constant proportion τ. So, if an item were discarded by the buffer at trial *j*, and *i* is the number of trials intervened between the original study and the test on that item, the amount of information stored in LTS at the time of test would be *jθτ*^*i*−*j*^.

In case of a subject undergoes a test on an item, the subject gives the correct response if the item is in the sensory or memory buffer, but if the item is not in either of these buffers, the subject searches LTS. This LTS search is called the *retrieval process*. In this regard, two important observations should be made: First, it is assumed that the likelihood of retrieving the correct response for a given item improves as the amount of information stored concerning that item increases. Second, the retrieval of an item gets worse; the longer the item has been stored in LTS. In other words, there is some sort of decay in information as a function of the length of time information has been stored in LTS.

After these assumptions and observations, it is then possible to specify the probability of a correct retrieval of an item from LTS. If the amount of information stored at the moment of test for an item is zero, then the probability of a correct retrieval should be at the guessing level. As the amount of information increases, the probability of a correct retrieval should increase toward unity. The authors define *p*_*ij*_ as the probability of a correct response from LTS of an item that had a lag of *i* trials between its study and test and that resided in the buffer for exactly *j* trials. Hence, such a probability can be mathematically written as


(6) $$\begin{array}{l} p_{ij}=1-\left(1-g\right)e^{-j\theta \tau^{i-j}} \end{array}$$

where *g* is the guessing probability; for example, if an experiment is made up of 26 response alternatives, then the guess probability is $\frac{1}{26}$.

#### 2.3.4 Strong and weak points of the Atkinson and Shiffrin model

##### 2.3.4.1 Strong points

Some of the strongness of the model can be summarized in the following way:

- It provides a good understanding of the structure and processes of the human memory.
- It is distinguished as it has generated a lot of research into memory.
- Many memory studies provide evidence to support the distinction between STM and LTM (in terms of encoding, duration, and capacity).
- Due to its multi-store structure, it is able to explain specific well-known case in neuropsychology, such as the case of Henry Gustav Molaison (Annese et al., 2014).

##### 2.3.4.2 Weak points

Despite the fact of being influential such a model has some weak points, we have those as follows:

- The model is oversimplified, for example, it suggests that each of the stores works as an independent unit, that is not the case.
- The model does not explain memory distortions (memory can be distorted when they are retrieved because there is a necessity to fill in the gaps to create meaningful memory).
- There are some memories that can be stored in long-term memory even if the amount of rehearsal is minimal, for example, a severe bicycle crash.
- Sometimes despite a prolonged rehearse action to remember information, it is not transferred to long-term memory.

#### 2.3.5 Mathematical developments

As already mentioned previously, the Atkinson-Shiffrin memory model is an influential model. It is no surprise to note that several models have been developed on its basis. In the following, we provide a chronological history of such developments.

The Search of Associative Memory (SAM) model by Raaijmakers and Shiffrin, was proposed in 1981 and described in Raaijmakers and Shiffrin (1981); the likelihood of remembering one of the remaining words is lower than if no cues are given at all when free recall of a list of words is prompted by a random subset of those words. SAM utilizes interword connections extensively in retrieval, a mechanism that has been overlooked by prior thinking, to predict this effect in all of its forms.

The SAM model for recall (Raaijmakers and Shiffrin, 1981) is extended by assuming that a familiarity process is used for recognition. The recall model, proposed in 1984 by Gillund and Shiffrin (1984), proposes probabilistic sampling and recovery from an associative network that is dependent on cues. The recall model postulates cue-dependent probabilistic sampling and recovery from an associative network. The recognition model, proposed by Gillund and Shiffrin, is strictly linked to the recall model because the total episodic activation due to the context and item cues is used in recall as a basis for sampling and in recognition to make a decision. The model predicts the results from a new experiment on the word-frequency effect.

In 1997, Shiffrin and Steyvers (1997), proposed the REM model (standing for retrieving effectively from memory) developed to predict places explicit and implicit memory, as well as episodic and general memory, into the framework of a more complex theory that is being created to explain these phenomena. The model assumes storage of separate episodic images for different words, each image consisting of a vector of feature values.

Mueller and Shiffrin (2006) presented the REM-II model, and this model is based on Bayesian statistics. REM-II models the development of episodic and semantic memory. Semantic information is represented by the model as a collection of these features' co-occurrences, while episodic traces are represented as sets of features with varying values. Feature co-occurrence approaches the complexity of human knowledge by enabling polysemy and meaning connotation to be recorded inside a single structure. The authors present how knowledge is formed in REM-II, how experience gives rise to semantic spaces, and how REM-II leads to polysemy and encoding bias.

The SARKAE (Storing and Retrieving Knowledge and Events) model proposed by Nelson and Shiffrin (2013), which represents a further development of the SAM model, describes the development of knowledge and event memories as an interactive process: Knowledge is formed through the accrual of individual events, and the storage of an individual episode is dependent on prior knowledge. To support their theory, the authors refer to two experiments that provide data to support the theory: These experiments involve the acquisition of new knowledge and then testing in transfer tasks related to episodic memory, knowledge retrieval, and perception

Lastly, we would like to point out that there are also models that are in contrast with the Atkinson and Shiffrin's original model; among these, there is a dynamic model by Cox and Shiffrin (2017), that consider that memory is cue-dependent, such a model is in line with MINERVA (see Section 2.5).

### 2.4 A neuromathematical model of human information

In 1983, Anderson (1983) proposed a neuromathematical model of human information processing. The acquisition of new contents is a fundamental part of cognition. Two fundamental aspects of such an acquisition are the rate of information processing during the learning phase and the efficiency of the subject (the learner) in mobilizing relevant information in long-term memory. They play a fundamental role in transmitting newly acquired information to stable storage in long-term memory. Hence, they are extremely important in (new) contents acquisition. In addition, these cognitive processes, moreover, may be substantially related in tempo and quality of organization to the efficiency of higher thought processes such as divergent thinking and problem-solving ability that characterize scientific thought. Being a critical topic in the study of memory, Anderson proposed and empirically evaluated a mathematical model of information acquisition.

According to Anderson, sufficient neuroscientific information is available to suggest that the processes of information acquisition in short-term memory (STM) can be modeled as a set of time-dependent equations representing rates of general processes in the central nervous system (CNS) activity.

#### 2.4.1 Stability function

Anderson assumed that the holding capacity of short-term memory is limited. Therefore, the stability of information in STM partially depends on the amount of information stored in STM and in general will decline as the information load increases. Some characteristics of the information could influence its efficient storage in STM and the capacity of the learner to effectively organize and transmit the information to long-term memory (LTM). Two properties of stimulus information considered by Anderson in this first approximation are (1) the information quality (β) and (2) the information quantity (ρ). Information quality is defined as the abstractness of the information. Then, Anderson introduces *S*, which represent the activity of the central nervous system associated with storage of information and its stability in short-term memory. The magnitude of this activity will decline as the load of information increases; in other words, the stability of information in STM decreases as STM holding capacity begins to reach saturating levels. The rate of decrease in stability with time will be proportional to the amount of activity accumulated. As a consequence, this mathematically is equivalent to write


(7) $$\begin{array}{l} -\frac{dS}{dt}=f\left(S\right). \end{array}$$

Equation (7) is the more general form for describing the rate of decrease in stability. Indeed, it should be considered that the rate of decrease in stability should be less for learners with higher intellectual ability than those of lower ability. Moreover, the rate of decrease in stability should be increased; the more abstract the information and the greater the rate of presentation (the larger the progression density). Both of these factors contribute to the cognitive demand placed on the learner. Hence, Anderson proposed the following refined statement that represents the instantaneous rate of change in stability:


(8) $$\begin{array}{l} -\frac{dS}{dt}=\frac{\alpha \beta \delta }{\kappa }S \end{array}$$

where α is a constant of proportionality, β is the content quality (i.e., the abstractness), δ is the content quantity (progression density), and κ is the learner's intelligence quotient properly scaled, are constant too. By integrating Equation (8) the analytical form of S is obtained:


(9) $$\begin{array}{l} S\left(t\right)=S_{0}e^{-\frac{\alpha \beta \delta }{\kappa }t} \end{array}$$

where *S*_0_ is the initial value of *S* at *t*_*o*_ and *t* is time since the start of the learning experience. This is a decreasing exponential function representing the rate of decay in stability of information in STM as information load increases with time. Equation (8) is, therefore, a time-dependent function representing CNS stability. In psychological terms, it is a prediction of the amount of residual short-term memory holding capacity at a point in time after onset of the learning experience. The amount of STM information storage capacity depends on the amount of information already stored in STM and the complexity of the incoming information as represented in part by the variables β and ρ in the rate coefficients of the equation. In addition, to the stability of information in STM, the amount of instability in CNS associated with uncertainty in encoding novel stimulus material must be considered.

#### 2.4.2 Instability function

As learning progresses and behavior becomes more differentiated, initial instability associated with the new learning task will decrease. Let *I* represent activity in the CNS associated with instability of the system and λ the coefficient of decay of *I* with time. Hence, the instantaneous rate of decay in instability of CNS for information encoding, which is related to the amount of activity *I* through the instability coefficient λ can be mathematically written as


(10) $$\begin{array}{l} \frac{dI}{dt}=-\lambda I \end{array}$$

The integration of Equation (10), with the initial condition *I*(*t* = 0) = *I*_0_, provides


(11) $$\begin{array}{l} I\left(t\right)=I_{0}e^{-\lambda t}. \end{array}$$

At any point in time, the capacity of the CNS to encode information will be equivalent to the difference between the stability function and the instability function or


(12) $$\begin{array}{l} S\left(t\right)-I\left(t\right)=S_{0}e^{-\frac{\alpha \beta \delta }{\kappa }t}-I_{0}e^{-\lambda t} \end{array}$$

Equation (12) represents the net encoding capacity of STM at an arbitrary point in time *t*.

#### 2.4.3 The gain function

Then, Anderson introduce CNS activity correlated with information gain, called *N*. Then, he wrote


(13) $$\begin{array}{l} \frac{dN}{dt}=\frac{\kappa }{\bar{\alpha }\beta \delta }N. \end{array}$$

In Equation (13), it is clear how the instantaneous rate of increase in information is directly proportional to N and κ, the intelligence of the subject, inversely related to β, and the abstraction of stimulus information, and δ, the progression density. $\bar{\alpha }$ is a constant of proportionality. Theoretically speaking, the gain function represents the amplification of CNS activity associated with the elaboration of information in memory through active memory processes of reorganization of information in LTM.

By solving the following Cauchy problem,


(14) $$\begin{array}{l} \{\begin{array}{c} \frac{dN}{dt}=\frac{\kappa }{\bar{\alpha }\beta \delta }N\\ N\left(0\right)=N_{0} \end{array} \end{array}$$

The obtained solution is


(15) $$\begin{array}{l} N\left(t\right)=N_{0}e^{\frac{\kappa }{\bar{\alpha }\beta \delta }t}. \end{array}$$

#### 2.4.4 Composite equation

The product of the gain function, Equation (15), and the modulation factor, Equation (12), yields the composite equation:


(16) $$\begin{array}{l} N_{t}\left(t\right)=N_{0}e^{\frac{\kappa }{\bar{\alpha }\beta \delta }t}\left(S_{0}e^{-\frac{\alpha \beta \delta }{\kappa }t}-I_{0}e^{-\lambda t}\right) \end{array}$$

where *N*_*t*_ is the net information's gain at time *t*. With appropriate choice of constants (α and $\bar{\alpha }$) and properly scaled variables (δ, β, κ, and λ), the equation yields learning curves that can be empirically tested in relation to data obtained in human learning experiments. The composite equation, therefore, represents the total information gain (*N*_*t*_) at a point in time (*t*) and is the product of the subjects' capacity to generate interrelationships among units of information in LTM (G factor), and the amount of immediate net STM encoding capacity (M factor).

#### 2.4.5 Strong and weak of Anderson's model

##### 2.4.5.1 Strong points

The model introduces differential equations to model human memory information processing in a simple form, immediately available to anyone. The model has yielded good predictions for student recall in short-term learning experiences.

##### 2.4.5.2 Weak points

The model is limited to cognitive phenomena in short-term learning experiences lasting on the order of minutes to one-half hour. It is based on the assumption that the subject (the learner) is not aided by external prompts such as notes or other forms of mental aids. Important factors such as the motivational state of the learner and/or fatigue and stress are not taken into account. A warning point (this holds for any model) is the duration of the learning experiences and the characteristics of the learners used in experimental studies: These parameters need to be carefully controlled to avoid biases that may be introduced if they deviate appreciably from a moderately motivated population.

#### 2.4.6 Mathematical developments

Anderson proposed some implemented versions of the original model. For example, in Anderson (1986), he included coefficients which represent the motivational state of the learner. In particular, two coefficients were included: the first is an exponential coefficient in the gain function representing largely a change rate of learning associated with varying motivation, while the second is an initial factor in the gain equation change in motivation at the outset of a learning task. This permits modeling of the effects of variations in motivation on the rate and amount of information in a learning task.

We remark that some criticisms to Anderson's model were moved by Preece and Anderson (1984). Preece suggests that Anderson's data could better, or “more parsimoniously”, represented by a learning model proposed by Hicklin (1976). In response to this critique, Anderson stated that several mathematical models have been created to forecast human learning curves, with a significant portion of these models being dependent on learner-specific characteristics. These models, however, do not take into account variations in the information input or the complexity of the information, such as the interaction between short- and long-term memory. Therefore, more complex models are required to explore more natural learning scenarios where information receipt occurs, and the Anderson model is designed to do just that.

### 2.5 MINERVA 2-A simulation model of human memory

In 1984, Hintzman (1984) proposed the so-called MINERVA 2-A simulation model of human memory. The model makes some assumptions: First, only episodic traces are stored in memory; second, repetition produces multiple traces of an item; third, a retrieval cue contacts all memory traces simultaneously; fourth, each trace is activated according to its similarity to the retrieval cue; five, all traces respond in parallel, the retrieved information reflecting their summed output. MINERVA 2 represents an attempt to account for data from both episodic and generic memory tasks within a single system. The theory underpinning the model is primarily concerned with long-term or secondary memory (SM) although it also assumes that there is a temporary working store or primary memory (PM) that communicates with SM. The interactions between the two stores are restricted to two elementary operations: PM can send a retrieval cue, or “probe”, into SM, and it can receive a reply, called the “echo.” When a probe is sent to SM, a single echo is returned. Information in the echo, and its relation to information in the eliciting probe, are the only clues available to PM regarding what information SM contains. The author remarks that SM is a vast collection of episodic memory traces, each of which is a record of an event or experience. An experience is assumed to occur when a configuration of primitive properties or features is activated in PM, and a memory trace is a record of such a configuration. The experience is strictly connected to a memory trace. Indeed, each experience leaves behind its own memory trace even if it is virtually the same as an earlier one. This means that the effects of repetition are mediated by multiple copies—or redundancy—rather than by strengthening. Hintzman speculates that there is no separate conceptual, generic, or semantic store. Hence, all information, whether specific or general, is retrieved from the pool of episodic traces that constitutes SM. When a probe is communicated from PM to SM, it is simultaneously matched with every memory trace, and each trace is activated according to its degree of similarity to the probe. The echo that comes back to PM represents the summed reactions of all traces in SM. In other words, there is no process by which individual memory traces can be located and examined in isolation. All SM traces are activated in parallel by the probe, and they all respond in parallel, and the echo contains their combined messages. A trace's contribution to the echo is determined by its degree of activation, so only traces that are relatively similar to the probe make a significant contribution to the echo.

#### 2.5.1 The model description

MINERVA 2 bears some similarity to MINERVA 1 (see Hintzman and Ludlam, 1980) but is applicable to a much wider variety of tasks. An experience (or event) is represented as a vector, whose entries (which represent the features, i.e., a configuration of primitive properties that activate so that an experience occurs) belongs to the set {+1, 0, −1}. The values +1 and −1 occur about equally often, so that over a large number of traces, the expected value of a feature is 0. In a stimulus or event description, a feature value of 0 indicates that the particular feature is irrelevant. In an SM trace description, a value of 0 may mean either that the feature is irrelevant or that it was forgotten or never stored. In learning, active features representing the present event are copied into an SM trace. Each such feature has probability *L* of being encoded properly, and with probability 1 − *L* the tract feature value is set at *O*. If an item is repeated, a new trace is entered into SM each time it occurs. The authors define *P*(*j*), it represents the feature *j* of a probe or retrieval cue, and *T*(*i, j*), a mathematical object (see Hintzman and Ludlam, 1980), which is the corresponding feature of memory trace *i*. *T*(*i, j*) must be statistically compared to *P*(*j*), that is why *T*(*i, j*) is a function both of the trace *i* and the probe *j*. The similarity of trace *i* to the probe is computed as


(17) $$\begin{array}{l} P\left(i\right)=\overset{N}{\underset{j=1}{\sum }}\frac{P\left(j\right)T\left(i,j\right)}{N}, \end{array}$$

where *N* is the total number of features that are nonzero in either the probe or the trace.

*S*(*i*) can be viewed a sort of correlation index: If *S*(*i*) = 0, then the probe and trace are orthogonal, if *S*(*i*) = 1, they perfectly match, taking on both positive and negative values. The activation level of a trace, *A*(*i*), is a positively accelerated function of its similarity to the probe. In the study's simulations,


(18) $$\begin{array}{l} A\left(i\right)=S{\left(i\right)}^{3}. \end{array}$$

Raising the similarity measure to the third power increases the *signal-to-noise* ratio, in that it increases the number of poorly matching traces required to overshadow a trace that closely matches the probe. It should be noted that if trace *i* was generated randomly (by a process orthogonal to that generating the probe), then the expected value of *A*(*i*) is 0 and the variance of *A*(*i*) is quite small. Thus, *A*(*i*) should be very near to 0 unless trace *i* fairly closely matches the probe.

##### 2.5.1.1 Intensity

When a probe activates the traces in SM, information is returned in the echo. The echo is assumed to have two properties: intensity and content. The intensity of the echo is given by


(19) $$\begin{array}{l} I_{E}=\overset{M}{\underset{i=1}{\sum }}A\left(i\right) \end{array}$$

where M is the total number of traces in memory. The variance of *I*_*E*_, *Var*(*I*_*E*_), is a function of the number of target traces. If *L* = 1, then this function is flat, reflecting only the baseline “noise” in *I* produced by non-target traces. If *L* < 1 and is constant, then *Var*(*I*_*E*_) increases linearly with frequency because the *A*(*i*) values of the individual target traces vary and contribute independently to *I*_*E*_. Frequency judgments and recognition judgments are assumed to be based on the intensity of the echo, and therefore, characteristics of the *I*_*E*_ distribution are crucial in simulating performance in these tasks.

##### 2.5.1.2 Content

The content of the echo is the activation pattern across features that is returned from memory following the probe. It is assumed that the activation of each SM trace, *i*, is passed to each of its constituent features, *j*, as the product of *A*(*i*) and *T*(*i, j*). Note that the product will be positive if the signs of *A*(*i*) and *T*(*i, j*) are the same and negative if they are different. The contributions of all *M* traces in memory are summed for each feature; thus, activation of feature *j* in the echo is given by


(20) $$\begin{array}{l} C\left(j\right)=\overset{M}{\underset{i=1}{\sum }}A\left(i\right)T\left(i,j\right). \end{array}$$

The values taken by *C*(*j*) can range from negative to neutral to positive, and their profile (i.e., the associated histogram) across features is assumed to be immediately available in PM. Only traces that are similar to the probe become strongly activated. The author remarks that those traces can contain information not present in the probe itself, and thus, the model is capable of associative recall.

In order to simulate the retrieval of associative information, the set of features can be divided into two segments. For example, to represent face-name pairs, features *j* = 1, ..., 10 might be reserved for the faces and the remaining features, *j* = 11, ..., 20, for the names. Then, a trace of 20 features would represent a single occurrence of a particular pair. Recall of a name upon presentation of a face can be accomplished with a probe having *j* = 1, ..., 10 filled in and *j* = 11, ..., 20 set to 0, focusing on *C*(11), ..., *C*(20) in the echo. Retrieval of a face given a name would be done in the opposite fashion.

#### 2.5.2 Strong and weak points of MINERVA 2

##### 2.5.2.1 Strong points

MINERVA 2 can deal with the problem of “ambiguous recall.” The ambiguous recall problem is that information retrieved from memory is sometimes only vaguely similar to what was originally stored or to any acceptable response.

##### 2.5.2.2 Weak points

The model is very simple and therefore limited in its applications.

#### 2.5.3 Mathematical developments

There is a rich literature regarding the developments as well as implementations of the Hinztman model. For example, the ATHENA model (see Briglia et al., 2018) as an enactivist^2^ mathematical formalization of Act-In model by Versace et al. (2014), within MINERVA2 non-specific traces: ATHENA is a fractal model which keeps track of former processes that led to the emergence of knowledge; in this way, it can process contextual processes (abstraction manipulation). An interesting characteristic of ATHENA is that it is a memory model based on an inference process that is able to extrapolate a memory from very little information (Tenenbaum et al., 2011). As a consequence, ATHENA accounts for the subjective feeling of recognition, unlike MINERVA2 (for details see Benjamin and Hirshman, 1998). As a final remark, it should be noted that Nelson and Shiffrin (2013) considered that this process should be implemented in SARKAE, as suggested and described by Cox and Shiffrin (2017).

### 2.6 Computational models of memory search

Kahana (2020) in his study reviewed the fundamental concepts in the mathematical modeling of human memory. We think it is worth analyzing them.

#### 2.6.1 Representational assumptions

The act of remembering involves accessing stored information from experiences that are no longer in the conscious present. In order to model remembering, it is necessary therefore define the representation that is being remembered. Mathematically, a static image can be represented as a two-dimensional matrix, which can be stacked to form a vector. Memories can also unfold over time, as in remembering speech, music, or actions. Although one can model such memories as a vector function of time, theorists usually eschew this added complexity, adopting a unitization assumption that underlies nearly all modern memory models. The unitization assumption states that the continuous stream of sensory input is interpreted and analyzed in terms of meaningful units of information. These units, represented as vectors, form the building blocks (units) of memory and both the inputs and outputs of memory models. Scientists interested in memory study the encoding, storage, and retrieval of these units of memory.

Let $\vec{f_{i}}\in {\mathbb{R}}^{N}$ represent the memorial representation (vector) of item *i* in the scalar space ℝ^*N*^. The *N* elements of the vector $\vec{f_{i}}$ are denoted by $f_{i}\left(1\right),\vec{f_{i}}\left(2\right),...,\vec{f_{i}}\left(N\right)$, that represent information in either a localist or a distributed manner. According to localist models, each item vector has a single, unique, non-zero element, with each element thus corresponding to a unique item in memory. Hence, the localist representation of item *i* can be viewed as a vector $\vec{f_{i}}\left(j\right)$, whose elements *f*_*i*_(*j*) are defined such that


(21) $$\begin{array}{l} f_{i}\left(j\right)=\{\begin{array}{cc} 0 & \text{ if }i\neq j\\ 1 & \text{ if }i=j \end{array} \end{array}$$

The last case represents the unit vectors.

Differently, according to distributed models, the features representing an item distribute across many or all of the elements. In this case, a probability *p* of assuming scalar 1 must be introduced. In detail, consider the case where *f*_*i*_(*j*) = 1 with probability *p* and *f*_*i*_(*j*) = 0 with probability 1 − *p*. The expected correlation between any two such random vectors will be zero, but the actual correlation will vary around zero. The same is true for the case of random vectors composed of Gaussian features as is commonly assumed in distributed memory models (see for example Kahana et al., 2005).

#### 2.6.2 Multitrace theory

Encoding is the set of processes where a subject (the learner) records information into memory. The subject does not simply record sensory images but, rather, creates the multidimensional (i.e., vectorial) representation of items as well as produce a lasting record of the vector representation of experience. To this aim, it needs to introduce another mathematical tool able to describe how the brain record a lasting impression of an encoded item or experience since the only vector is not enough to do that. Such a mathematical tool is the matrices. Mathematically, the set of items in memory form a matrix, that is basically an array, where each row represents a feature or dimension, and each column represents a distinct item occurrence. The matrix encoding item vectors $\vec{f_{1}},\vec{f_{2}},\vec{f_{3}},...,\vec{f_{t}}$ can be represented as follows:


(22) $$\begin{array}{l} \bar{\bar{M}}=\left[\begin{array}{cccc} f_{1}\left(1\right) & f_{2}\left(1\right) & \cdots & f_{t}\left(1\right)\\ f_{1}\left(2\right) & f_{2}\left(2\right) & \cdots & f_{t}\left(2\right)\\ \vdots & \vdots & \vdots & \vdots \\ f_{1}\left(N\right) & f_{2}\left(N\right) & \cdots & f_{t}\left(N\right) \end{array}\right] \end{array}$$

where the first column of the matrix represents the entries (i.e., the elements) of vector $\vec{f_{1}}$, the second column the entries of vector $\vec{f_{2}}$ and so on. The multitrace hypothesis implies that the number of traces can increase without bound. In summary, the multitrace theory positing that new experiences, also including repeated ones, add more columns to the growing memory matrix $\bar{\bar{M}}$ described in Equation (22). Nevertheless, without positing some form of data compression, the multitrace hypothesis creates a formidable problem for theories of memory search.

#### 2.6.3 Composite memories

This theory, in contrast with the view that each memory occupies its own separate storage location, states that memories blend together in the same manner that pictures may be combined (as happens in morphing). From a mathematical point of view, this translates in simply summing the vectors representing each image in memory. Then, there are at least two techniques to be used to deal with such a sum: first, averaging the sum of features, but in this way, information about the individual exemplars are discarded; second, defining a composite storage model to account for data on recognition memory, as proposed by Murdock (1982). This model specifies the storage equation in the following way:


(23) $$\begin{array}{l} \vec{m_{t}}=\alpha \vec{m_{t-1}}+\bar{\bar{B_{t}}}\vec{f_{t}}, \end{array}$$

where $\vec{m_{t}}$ is the memory vector and $\vec{f_{t}}$ represents the item studied at time *t*. The variable 0 < α < 1 is a forgetting parameter, and ${\bar{\bar{B}}}_{t}$ is a diagonal matrix whose entries ${\bar{\bar{B}}}_{t}\left(i,i\right)$ are independent Bernoulli random variables (i.e., a variables that take the value of 1 with probability *p* and 0 with probability 1 − *p*). The model parameter, *p*, determines the average proportion of features stored in memory when an item is studied.

If the same item is repeated, then it is encoded again. Indeed, some of the features sampled on the repetition could not be previously sampled; hence, repeated presentations will fill in the missing features, thereby differentiating memories and facilitating learning. It is possible to consider the feature of the studied items as independent and identically distributed normal random variables as done by Murdock (1982).

Rather than summing item vectors directly, it is better first expanding an item's representation into a matrix form and then sum the resultant matrices since if not there would be a substantial loss of information. Although this is beyond the scope of this study, we note that this operation forms the basis of many neural network models of human memory (Hertz et al., 1991). In this case, the entries of vector $\vec{f}$ represent the firing rates of neurons, then the vector outer product $\vec{f}\cdot \vec{f^{T}}$ forms a matrix $\bar{\bar{M}}$ whose entries are ${\bar{\bar{M}}}_{i,j}=f\left(i\right)f\left(j\right)$. Incidentally, this matrix exemplifies the Hebbian learning. However, this treatment could be interpreted as oversimplified since Hopfield network is not considered. The matrix $\bar{\bar{M}}$ should represent connections between neurons in the network, which itself defines transitions of the network state, and the fixed point of the dynamic is desired memory. We refer interested readers to Hopfield (2007) and related references.

#### 2.6.4 Summed similarity

If an item has already encoded and it is encountered again, we often quickly recognize it as being familiar. To create this sense of familiarity, the brain must somehow compare the representation of the new experience with the contents of memory. Such a research could be lead in series or in parallel. In the former case, the target item is compared to each stored item memory until a match is found. This process is generally slow. In the latter case, the research is in parallel, meaning by this that a simultaneous comparison of the target item with each of the items in memory. This second process is faster. Nevertheless, there is a point of attention to be considered: when an item is encoded in different situations, the representations will be very similar but not identical. Summed similarity models present a potential solution to this problem. Rather than requiring a perfect match, we compute the similarity for each comparison and sum these similarity values to determine the global match between the test probe and the contents of memory. There are a few similarity models, one of the simplest summed-similarity model is the recognition theory first proposed by Anderson (1970) and finally elaborated by Murdock (1989). The model elaborated by Murdock is called TODAM (Theory of Distributed Associative Memory). In this model, subjects store a weighted sum of item vectors in memory as detailed in Equation (23). In order to establish if a (test) item was already encoded, it is necessary that the dot product between the vector characterizing the item and the memory vector exceeds a threshold. Specifically, the model states that the probability of finding a perfect match (we denote this case with “OK”) between the test item (called $\vec{g}$) and one of the stored memory vectors is


(24) $$\begin{array}{l} P\left(OK\right)=P\left(\vec{g}\cdot \vec{m_{t}}>k\right)=P\left(\vec{g}\cdot \left[\overset{L}{\underset{t=1}{\sum }}\alpha^{L-t}{\bar{\bar{B}}}_{t}\vec{f_{t}}\right]>k\right) \end{array}$$

The TODAM embodies the direct summation model of memory storage. Such a summation model of memory storage implies that memories form a prototype representation. Hence, each individual memory contributes to a weighted average vector whose similarity to a test item determines the recognition decision. However, some criticisms are moved to this approach. Indeed, studies of category learning indicate that models based on the summed similarity between the test cue and each individual stored memory provide a much better fit to the empirical data than do prototype models (Kahana and Bennett, 1994). Some alternative approaches (see for example Nosofsky, 1992) represent psychological similarity as an exponentially decaying function of a generalized distance measure. That is, they define the similarity between a test item, $\vec{g}$, and a (fixed) studied item vector, $\vec{f_{i^{*}}}$, where i^*^ is any fixed value between 1 and *L*, as


(25) $$\begin{array}{l} Similarity\left(\vec{g},\vec{f}\right)=e^{-\tau \|\vec{g}-\vec{f_{i^{*}}}{\|}_{\gamma }}=e^{-\tau {\left[\sum_{j=1}^{N}{\left[g\left(j\right)-f_{i^{*}}\left(j\right)\right]}^{\gamma }\right]}^{\frac{1}{\gamma }}}, \end{array}$$

where *N* is the number of features, γ indicates the distance metric (γ = 2 corresponds to the Euclidean norm), and τ determines how quickly similarity decays with distance. Equation (25) can be generalized to *L* items, by considering the encoding item vectors $\vec{f_{i}}$, *i* = 1, ..., *L* vectors and the corresponding memory matrix $\bar{\bar{M}}=\left(\vec{f_{1}},\vec{f_{2}},\vec{f_{3}},...,\vec{f_{L}}\right)$. Then, the generalized equation is obtained by summing the similarities between $\vec{g}$ and each of the stored vectors in memory,


(26) $$\begin{array}{l} S=\overset{L}{\underset{i=1}{\sum }}Similarity\left(\vec{g},\vec{f_{i}}\right). \end{array}$$

The summed-similarity model generates an “OK” match if *S* exceeds a threshold.

We remark that $\vec{g}$ can play the role either of target (i.e., $\vec{g}=\vec{f_{i}}$ for some value of *i*) or probe, in this last case $\vec{g}\notin \bar{\bar{M}}$.

#### 2.6.5 Contextual coding

Another relevant point in the study of memory encoding is temporal coding, associations are learned not only among items but also between items and their situational, temporal, and/or spatial context (see for example, some fundamental studies such as Carr, 1931). The idea of temporal coding was developed more recently in 1970 by Tulving and Madigan (1970). Specifically, these authors distinguished temporal coding from contemporary interpretations of context. Differently from this, subsequent research brought these two views of context together: this is the case shown in Bower's temporal context model (Bower, 1972). According to Bower's model, contextual representations constitute a multitude of fluctuating features, defining a vector that slowly drifts through a multidimensional context space. These contextual features form part of each memory, combining with other aspects of externally and internally generated experience. Because a unique context vector marks each remembered experience, and because context gradually drifts, the context vector conveys information about the time in which an event was experienced. By allowing for a dynamic representation of temporal context, items within a given list will have more overlap in their contextual attributes than items studied on different lists or, indeed, items that were not part of an experiment (see Bower, 1972). It is possible to implement a simple model of contextual drift by defining a multidimensional context vector, $\vec{c}=\left[c\left(1\right),c\left(2\right),...,c\left(N\right)\right]$, and specifying a process for its temporal evolution. To this aim, it needs specify a unique random set of context features for each list in a memory experiment or for each experience encountered in a particular situational context. However, contextual attributes fluctuate as a result of many internal and external variables that vary at many different timescales. An alternative approach proposed by Murdock (1997), is to write down an autoregressive model for contextual drift, such as


(27) $$\begin{array}{l} \vec{c_{i}}=\vec{c_{i-1}}+\sqrt{1-\rho^{2}}\vec{\epsilon } \end{array}$$

where $\vec{\epsilon }$ is a random vector whose elements are each drawn from a Gaussian distribution, while each item presentation is represented by *i* indexes. The variance of the Gaussian is defined such that the inner product $\vec{\epsilon_{i}}\cdot \vec{\epsilon_{i}}$ equals one for *i* = *j* and zero for *i* ≠ *j*. Accordingly, the similarity between the context vector at time steps *i* and *j* falls off exponentially with the separation: $\vec{c_{i}}\cdot \vec{c_{i}}=\rho^{\left|i-j\right|}$. This means that the change in context between the study of an item and its later test will increase with the number of items intervening between the study and the test, producing the classic forgetting curve. In terms of the study of memory and in continuity with the above sections, it is possible to concatenate each item vector with the vector representation of context at the time of encoding (or retrieval) and store the associative matrices used to simulate recognition and recall in our earlier examples. An alternative way is directly associate context and item vectors in the same way that we would associate item vectors with one another.

#### 2.6.6 Strong and weak points of the models

##### 2.6.6.1 Strong points

The above described models are based on mathematics and linear algebra. In this sense, they are definitely innovative. One immediate consequence is that a computation approach, we mean the creation of codes can be naturally implemented.

##### 2.6.6.2 Weak points

The models show a main limitation: They cannot explain diseases affecting episodic memories. In order to bypass this criticism, it needs to modify their analytical form.

#### 2.6.7 Mathematical developments

These models are quite recent, therefore, as far as we know, there are no developments published in the literature yet.

### 2.7 Conclusion and future challenges

Modeling and computation are intended to take on an increasingly important role in (neuro)psychology, neuroscience, and psychiatry. One of the most important consequences of the mathematical modeling of human memory is to better understand the diseases affecting it. Modeling such diseases and find computational biomarker could also represent a great help to (neuro)psychologists and physicians. As a final step, we shortly describe the most relevant memory diseases whose distinctive traits, such as amnesias, could be mathematically modeled.

#### 2.7.1 Alzheimer's disease (AD)

Maybe Alzheimer's disease (AD) is the most popular neurological disease affecting memory (Eustache et al., 1990), and the most common form of dementia (Jack, 2012). It is a progressive, degenerative, and fatal brain disease, in which synapses connections in the brain are lost. The evidence suggests that women with AD display more severe cognitive impairment relative to age-matched males with AD as well as a more rapid rate of cognitive decline (Dunkin, 2009).

#### 2.7.2 Semantic dementia (SD)

Semantic dementia (SD) designates a progressive cognitive and language deficit, primarily involving comprehension of words and related semantic processing, as described in a very pioneering work by Pick (1904). These patients lose the meaning of words, usually nouns, but retain fluency, phonology, and syntax. Semantic dementia is distinguishable from other presentations of frontotemporal dementia (see Section 2.7.3) and Alzheimer's disease (see Section 2.7.1) not only by fluent speech and impaired comprehension without the loss of episodic memory, syntax, and phonology but also by empty, garrulous speech with thematic perseverations, semantic paraphasias, and poor category fluency.

#### 2.7.3 Fronto-temporal dementia (FTD)

Frontotemporal dementia is an uncommon type of dementia that causes problems with behavior and language. It is result of damage to neurons in the frontal and temporal lobes of the brain. Many possible symptoms can result, including unusual behaviors, emotional problems, trouble communicating, difficulty with work, or difficulty with walking.

#### 2.7.4 A case study: autobiographical amnesia

Talking about neurodegenerative diseases one relevant case of interest is autobiographical amnesia (Piolino et al., 2003). There are different theories regarding long-term memory consolidation that can be applied to investigate pathologies involving memory. For example, according to the standard model of systems consolidation (SMSC) (Squire and Alvarez, 1995), the medial temporal lobe (MTL) is involved in the storage and retrieval of episodic and semantic memories during a limited period of years. An alternative model of memory consolidation, called the multiple trace theory (MTT), posits that each time some information is presented to a person, it is neurally encoded in a unique memory trace composed of a combination of its attributes (Semon, 1923). In other words, it suggests that the capacity of the MTL to recollect episodic memories is of a more permanent nature. Piolino et al. (2003), to test these models, studied three groups of patients with a neurodegenerative disease predominantly affecting different cerebral structures, namely, the MTL (patients in the early stages of Alzheimer's disease) and the neocortex involving either the anterior temporal lobe (patients with semantic dementia) or the frontal lobe (patients with the frontal variant of frontotemporal dementia, fv-FTD). Then, they compared these groups of patients (the cardinality of the three set of patients was nearly the same) with control subjects using a specific autobiographical memory task designed specially to assess strictly episodic memory over the entire lifespan.

This task considers the ability to mentally travel back in time and re-experience the source of acquisition by means of the remember/know paradigm. The outcome was interesting since all three groups of patients produced strongly contrasting profiles of autobiographical amnesia regardless of the nature of the memories in comparison with that of the control group. In details, temporally graded memory loss in Alzheimer's disease, showing that remote memories are better preserved than recent ones; in semantic dementia, memory loss is characterized by a reversed gradient,^3^ while memory loss without any clear gradient was found in fv-FTD. By focusing on episodic memories (see Section 1), the authors found that they were impaired, whatever the time interval considered in the three groups, though the memory loss was ungraded (i.e., no temporal gradient was detected) in Alzheimer's disease and fv-FTD and temporally graded in semantic dementia, sparing the most recent period.^4^ A deficit of autonoetic consciousness^5^ emerged in Alzheimer's disease and fv-FTD but not in semantic dementia though beyond the most recent 12-month period. The authors remarked that the sematic dementia group could not justify their subjective sense of remembering to the same extent as the controls since they failed in providing contextual information, spatial or temporal details, etc. The results demonstrated that autobiographical amnesia varies according to the nature of the memories under consideration and the locus of cerebral dysfunction. The analysis was carried on by considering both the two competing models for long-term memory consolidation above described (i.e., SMSC and MTT), the authors observed that new insights based on concepts of episodic memories in the early of 2000s challenge the standard model and tend to support the MTT instead.

##### 2.7.4.1 How the mathematical models could face (autobiographical) amnesia

After having introduced the autobiographical amnesia, we would like to provide the reader with an example of how amnesia can be differently modeled by employing some models, as well as their implementations, above described. A first approach is based on the Ribot's law, and its implementation (Murre et al., 2013). Murre et al. hypothesized the decline function as an exponential function characterized by a constant decay rate even if it should be observed that the exponential decline assumption is not critical for the working of the model. The relation between memory intensity and recall probability can be described by a simple function:


(28) $$\begin{array}{l} p\left(t\right)=1-e^{-intensity\left(t\right)} \end{array}$$

Typically, a forgetting function is characterized by the fact that the “hippocampus” process declines rapidly, while the “neocortex” process builds up intensity. The neocortical process builds up slowly and eventually comes to a halt when the hippocampus process is depleted. There are two parameters that define the model: the first parameter relates to how quickly newly created traces fill up a process. The decline rate, which the authors designate as *a*_1_ and *a*_2_ for the neocortex and hippocampal regions, respectively, is the second parameter. Conversely, μ_1_ and μ_2_ denote the intensity gained during learning (the hippocampus plays a role in this process) and the rate at which consolidation fills the neocortex, respectively.

The Ribot gradient (see Section 2.2), i.e., the temporal gradient in retrograde amnesia, is characterized by a pattern with disproportional memory loss for recent time periods. Murre et al. made the hypothesis that the hippocampal, as well as the adjacent medial temporal lobe (MTL), process is damaged in amnesia. In this case, the contribution of the hippocampal and MTL processes are removed. In the memory chain model proposed by the authors, the total memory intensity, *r*(*t*) is the sum of the intensities of two processes: *r*_1_(*t*), the intensity of the hippocampal process, and *r*_2_(*t*), the intensity of the neocortical process. Hence,


(29) $$\begin{array}{l} r\left(t\right)=r_{1}\left(t\right)+r_{2}\left(t\right) \end{array}$$

It should be noted the time dependence in Equation (29). Indeed, a full lesion at time *t*_*l*_ of the hippocampus translates to removing the contribution of *r*_1_(*t*_*l*_) from the total intensity *r*(*t*_*l*_). In such a case, the neocortical intensity, *r*_2_(*t*_*l*_), which reflects the result of the consolidation process until the lesioning time *t*_*l*_, is the only term surviving. The authors remarked that tests of retrograde amnesia do not measure intensity directly but they rather measure recall probability. The predicted shape of these test gradients is, therefore, given by the following equation:


(30) $$\begin{array}{l} p_{Ribot}\left(t\right)=1-e^{-r_{2}\left(t_{l}\right)} \end{array}$$

If the hippocampus is lesioned at time *t*_*l*_, then there no more memories will be formed after that. There will also be no more consolidation from hippocampus-to-cortex. We have already explained in Section 2.2.2, the consequences and how Equation (30) changes.

Another approach addresses to the Atkinson and Shiffrin model (Atkinson and Shiffrin, 1968). In Section 2.3.3, we have described the mathematical formalization of the model. In case of amnesia, we expected that the information, which is transferred to LTS at a constant rate θ, changes since θ does. In our opinion, θ reduces though it does not necessarily vanish, apart from serious cases where memory circuits are permanently broken. The most relevant impact interests the *retrieval process*. Such a process degrades since it is assumed that the likelihood of retrieving the correct response for a given item improves as the amount of information stored concerning that item increases. As already introduced, see Section 2.3.3, the probability of a correct response from LTS of an item that had a lag of *i* trials between its study and test, and that resided in the buffer for exactly *j* trials. Hence, such a probability can be mathematically written as


(31) $$\begin{array}{l} p_{ij}=1-\left(1-g\right)e^{-j\theta \tau^{i-j}}, \end{array}$$

where *g* is the guessing probability. In case of amnesia, we expect that *g* approaches 0 and that θ became smaller and smaller depending on the degree of severity of amnesia. In the most extreme case, θ tending toward zero, *p*_*ij*_ vanishes.

These approaches are really different. In our opinion, they have pros and cons. For example, the approach by Murre et al. is really interested by a mathematical point of view. The idea to consider the hippocampus and neocortex as “big players” in amnesia is embraceable. However, they are not the only cerebral areas of interest in this kind of disease, just think about the thalamus. Furthermore, the same conclusions could be drawn by considering other analytical functions different from the exponential. Regarding the Atkinson and Shiffrin approach, the strong point is a statistical approach. Similarly to the previous case, such approach can well describe the case of partial or total hippocampus removal (see for example the case of Henry Gustav Molaison, also known as “Patient H.M.”^6^). By using this model, we cannot take into account factors such as motivation, effect and strategy (e.g., mnemonics techniques).

### 2.8 Final remark

The case study above described is just an example, other conditions such as chronical stress have also tremendously impact on human memory. Mathematical modeling could be an efficient tool to shed more light on it, as well as on other mnemonic pathologies.

## Author contributions

PF: Conceptualization, Investigation, Methodology, Resources, Writing—original draft, Writing—review & editing. FE: Conceptualization, Investigation, Methodology, Supervision, Validation, Writing—original draft.

## Footnotes

^1^We recall that a in cognitive psychology the chunking is a process by which small individual pieces of a set of information, the chunks, are bound together to create a meaningful whole later on in memory (Miller, 1956). Nevertheless, short-term memory is limited in capacity, consequently it severely limits the amount of information that can be attended to at any one time.

^2^Enactivism is a theory describing cognition as a mental function that arises from the dynamic interaction of the organism with its environment.

^3^In cognitive psychology a reverse temporal gradient denotes a pattern of retrograde amnesia characterized by greater loss of memory for events from the recent past (i.e., close to the onset of the amnesia) than for events from the remote past.

^4^Retrograde amnesia is usually temporally graded, which means that the most recent memories are affected first, and your oldest memories are usually spared. This is known as Ribot's law, see Section 2.2.

^5^Autonoetic consciousness is the human ability to mentally place oneself in the past and future (i.e., mental time travel) or in counterfactual situations (i.e. alternative outcomes), and to thus be able to examine one's own thoughts.

^6^Patient H.M. is an important case study in (neuro)psychology. Indeed, a large portion of his hippocampus was removed during a surgery to alleviate severe epilepsy.He was left with anterograde amnesia, but completely unable to form new explicit memories. This case was crucial to understand the role of the hippocampus in memory formation.
